# P01.55. Turmeric inhibits parathyroid hormone-related protein (PTHrP) secretion from human rheumatoid synoviocytes

**DOI:** 10.1186/1472-6882-12-S1-P55

**Published:** 2012-06-12

**Authors:** J Frye, B Timmermann, J Funk

**Affiliations:** 1 University of Kansas, Lawrence, USA; 2 University of Arizona, Tucson, USA

## Purpose

Excessive production of parathyroid hormone-related protein (PTHrP) by tumor-like synoviocytes contributes to joint destruction in rheumatoid arthritis (RA). Having previously demonstrated that curcuminoid-only and essential oil-only fractions of turmeric prevent joint destruction in an animal model of RA, we hypothesized that synoviocyte PTHrP production could be one signaling pathway targeted by turmeric (Curcuma longa L.) in RA.

## Methods

Two turmeric extracts were isolated from dried turmeric rhizomes and chemically characterized by HPLC, an essential oil-free curcuminoid-containing fraction and an essential oil-only fraction. Their effects on IL-1beta stimulated PTHrP secretion from human rheumatoid synoviocytes were assessed using a commercial PTHrP ELISA and primary cultures of synoviocytes isolated from patients with RA, as defined by the American College of Rheumatology.

## Results

Both turmeric fractions inhibited IL-1 stimulated PTHrP secretion from human rheumatoid synoviocytes in a dose-dependent fashion. The curcuminoid-containing fraction, which had no effect on constitutive PTHrP secretion, inhibited IL- stimulated PTHrP secretion with a least effective concentration (LEC) of 3 ug/ml. The essential oils, while slightly but significantly increasing constitutive PTHrP secretion, were potent suppressors of IL-1 stimulated PTHrP secretion (LEC = 1 μg/ml).

## Conclusion

Curcuminoids and essential oils of turmeric are both potent suppressors of cytokine-stimualted PTHrP secretion from human rheumatoid synoviocytes. When coupled with our previous *in vivo* studies demonstrating protective effects of both extracts in an animal model of rheumatoid arthritis, these findings suggest that both secondary metabolites of turmeric may have medicinal effects in the treatment of rheumatoid arthritis.

